# Prescription practices for malaria in Mozambique: poor adherence to the national protocols for malaria treatment in 22 public health facilities

**DOI:** 10.1186/s12936-015-0996-5

**Published:** 2015-12-01

**Authors:** Cristolde A. Salomão, Jahit Sacarlal, Baltazar Chilundo, Eduardo Samo Gudo

**Affiliations:** National Institute of Health, Ministry of Health, Field Epidemiology and Laboratory Training Program, Av Eduardo Mondlane 1008, 2nd floor, PO Box 264, Maputo, Mozambique; Department of Microbiology, Faculty of Medicine, Eduardo Mondlane University, Av. Salvador Allende 702, PO Box 257, Maputo, Mozambique; Department of Community Health, Faculty of Medicine, Eduardo Mondlane University, Av. Salvador Allende 702, PO Box 257, Maputo, Mozambique

**Keywords:** Malaria management, Over-treatment of malaria vs under-diagnosis of test-based malaria, Over-treatment of malaria

## Abstract

**Background:**

Current World Health Organization and national protocols recommend the ‘test and treat’ strategy for the management of uncomplicated malaria, to reduce over prescription of artemisinin-based combination treatment (ACT). Therefore, adherence to these protocols varies in different sub-Saharan African countries and no information is available for Mozambique. This study was conducted with the aim to evaluate the prescription practices of ACT in Mozambique.

**Methods:**

Retrospective audit of medical records corresponding to the period between July and December 2011 was conducted in 22 health units across 11 provinces in Mozambique. Two health units were selected per province according to availability of laboratory data (performing microscopy and rapid diagnostics testing-RDT or RDT only) and geographic setting (rural versus urban). At each facility, demographic data, laboratory results (blood smear or RDT), and prescription of ACT were all collected from the existing records.

**Results:**

Between July and December 2011, a total of 61,730 cases were tested for malaria, of which 42.7 % (26,369/61,730) were positive. A total of 35.361 patients were malaria negative, and ACT was prescribed to 72.0 % (25.448/35.361) of them. Prescription of ACT to malaria negative patients was higher in the central region of the country as compared to the northern and southern (81.1 % in the central region versus 72.4 and 63.7 % in the northern and southern, respectively, p = 0.000) and in urban settings (88.7 % in rural versus 58.0 % in urban settings, p = 0.000). Stock out of RDT was observed in six (27.3 %) of the health facilities. When no RDT was available, patients were empirically treated with ACT.

**Conclusion:**

Findings from this study demonstrate that health care worker’s adherence to the new guidelines for malaria treatment is poor in Mozambique and prescription of ACT to malaria negative patients remains very high. Enhanced training and supervision activities, community education and external quality assurance might lead to significant improvements in the clinician’s adherence to the new guideline for malaria treatment in Mozambique.

## Background

Accurate and timely diagnosis and treatment of malaria are critical for proper management of the disease and other treatable febrile illness. Presumptive diagnosis and treatment of malaria, with no reliance on laboratory confirmation, was for a long time the common practice in sub-Saharan Africa, including Mozambique [1–3]. While ineffective, this leads to over-diagnosis of malaria, over-treatment with anti-malarials, rapid emergence of drug-resistant strains and increased mortality among febrile patients because the true aetiology remains untreated [4–6].

In 2010, the World Health Organization (WHO) changed the recommendation for the management of uncomplicated malaria from presumptive diagnosis to the ‘test and treat’ strategy. As per this guidance, prescription of artemisinin-based combination treatment (ACT) should be entirely based on laboratory confirmation, either by microscopy or rapid diagnostic test (RDT), where this is available [7]. Most African countries, including Mozambique, adopted this recommendation and are rapidly scaling up RDT to areas where microscopy is not available [8]. Implementation of this recommendation, will not only avoid unnecessary prescription of ACT to non-malaria cases, but will also improve the management of non-malaria febrile illnesses and will delay the occurrence of drug-resistance strains [9]. Despite the rapid expansion of microscopy and widespread availability of malaria RDT, adherence to the guidelines for malaria treatment is still a challenge in sub-Saharan Africa, where administration of ACT to malaria negative patients is still high [10–12]. For instance, a recent study conducted in Tanzania, found that up to 90 % of anti-malarial drugs were prescribed to patients with negative malaria results [13]. Zurovac et al. [14] investigated the financial aspects of malaria management and found that adherence to the guidelines for malaria treatment might save up to 50 % of the costs of malaria programmes in sub-Saharan Africa. Poor adherence to the guidelines for malaria treatment in sub-Saharan Africa has been attributed to insufficient training, lack of supervision, socio-cultural aspects and lack of trust in the laboratory systems [15, 16]. To data, no study has been conducted to investigate the magnitude of this problem in Mozambique.

Although health care workers’ adherence to the new malaria guidelines has been extensively investigated in sub-Saharan Africa, data from different countries has not shown a consistent pattern. While significant improvement in the management of malaria was achieved in several countries [9, 17, 18], poor adherence to the guidelines for malaria was observed in others [10, 19].

This debate has been renewed in the light of the recent decline in malaria transmission in several parts of sub-Saharan Africa [20–23]. It is conceivable that reduction of malaria attributable fever, may increases over-prescription of ACT. With regards to Mozambique, previous reports demonstrate that malaria prevalence is in decline, given national efforts to control the disease [20, 24, 25], and also because of the implementation of the Lubombo Spatial Development Initiative and Roll Back Malaria activities [25–28].

Despite these gains, no study has yet been conducted in Mozambique to investigate the clinician’s adherence to the new malaria management protocols. This study was conducted to assess the prescription practices among health care workers in 22 health facilities in Mozambique. Results of this study will guide the National Malaria Control Programme (NMCP) in Mozambique to improve malaria case management in the country.

## Methods

### Study area and design

**Fig. 1:**
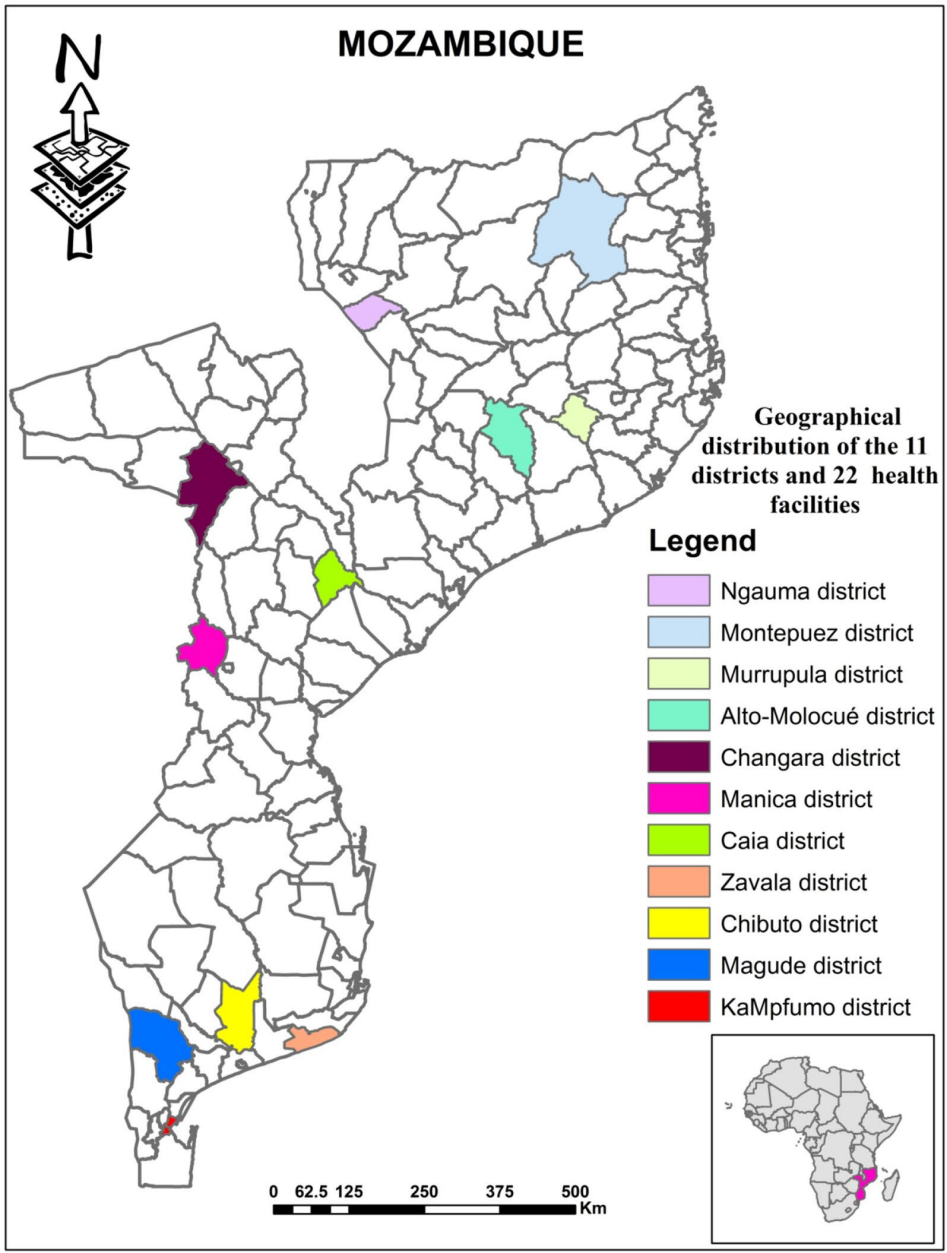
Geographical distribution of 11 districts and 22 health facilities. *Coloured districts* represent the selected districts in each province

Retrospective data from July to December 2011 was collected in 22 health facilities throughout the Mozambique’s 11 provinces. In each province, one district was selected from which two health facilities were then selected (Fig. 1). Districts and health facilities were selected based on the following criteria: (i) accessibility: only districts that were accessible by road were selected, (ii) geographical localization: two health facilities were selected in each district, one situated in an urban area and another situated in a rural area, and (iii) availability of laboratory facilities: of the two health facilities selected per district, one had laboratory facility (performing both microscopy and RDT) and another did not (performing only RDT).

Mozambique is situated on the southeast coast of Africa, with a total area of 801,590 square km and over 2500 km of coastline. The climate is typically tropical with two distinct seasons, the rain season from October to March and the dry season from April to September. The relative humidity is high and ranges between 70 and 80 %. The average annual precipitation is estimated to be 600 mm, and varies between 500 and 900 mm. The total population of the country is estimated to be 27 million, of which approximately 70 % lives in rural areas. Smallholder agriculture and fishing represents the main source of income [29, 30]. The country is administratively divided into three regions (north, center and south), has 11 provinces and 128 districts. Malaria is holoendemic in Mozambique, accounting for an estimated 48 % of all outpatient visits and nearly 63 % of all paediatric admissions. It is also estimated that malaria accounts for 26 % of total mortality in the country [31]. Mozambique is highly vulnerable to natural disasters such as droughts, cyclones and floods that often contribute to increased transmission of malaria.

### Ethics statement

This study was approved by the Institutional Bioethics Committee (ref#: 009.1/CIBS-INS/2012) prior to study initiation.

### Data collection

Experienced health workers from the Provincial Health Directorates reviewed the medical records and collected data using a standardized questionnaire. At each health facility, the laboratory results were collected from the laboratory logbook. Prescription information and information on RDT and ACT stock out were obtained from the pharmacy logbook. Prior to study initiation, each data abstractor received a comprehensive training on how to collect each information properly. The study researchers were responsible for the quality control of the questionnaires.

### Data analysis

Data were entered into a database developed using Epi info v 3.5.4 (2008). Each questionnaire was entered in duplicate and data matching was performed to identify errors during data entry. Duration of RDT and ACT stock out was dichotomized into “partial stock out” and “total stock out,” where stock out occurring for less than 30 days in a 1 month period was defined as partial, while stock out occurring during the entire month was defined as total. Analysis was performed using SPSS statistical package, version 17 (IBM Corp., Armonk, NY, USA), to calculate the frequencies, proportions and bi-variate analysis.

## Results

### General characteristics of the study sample

Between July and December 2011, a total of 61,730 patients were tested for malaria across 22 health facilities. Of these, 26,369 (42.7 %) were positive. Table 1 shows that the proportion of patients with positive malaria results was higher in health units without laboratory facilities (59.4 %; 4650/7826) as compared to those with laboratory facilities (40.3 %; 21,719/53,904), slightly higher in health facilities located in the northern region (54.1 %; 8535/15,783) as compared to those situated in the central (37.9 %; 8021/21,172) and southern (39.6 %, 9813/24,775) region of the country, and higher in health facilities located in rural settings (49.8 %; 15,891/31,896) as compared to those situated in urban settings (35.1 %, 10,478/29,834).

**Table 1:** Frequency of patients tested, confirmed and treated with ACT stratified by type of health facility

Classification of health facility	A—tested for malaria	B—malaria pos result (B/A %)	C—malaria neg result	D—patients treated with ACT	E—patients with malaria neg treated with ACT, n (E/C %)	*p* value
Availability of laboratory at the health facility						
With laboratory (microscopy and RDT)	53.904	21.719 (40.3)	32.185	44.883	23.164 (72.0)	0.899
Without laboratory (RDT)	7.826	4.650 (59.4)	3.176	6.934	2.284 (71.9)	
Geographical region						
North	15.783	8.535 (54.1)	7248	13.782	5.247 (72.4)	0.000
Center	21.172	8.021 (37.9)	13151	18.694	10.673 (81.1)	
South	24.775	9.813 (39.6)	14.962	19.341	9.528 (63.7)	
Location of health facility						
Urban	29.834	10.478 (35.1)	19.356	21.703	11.225 (58.0)	0.000
Rural	31.896	15.891 (49.8)	16.005	30.114	14.223 (88.7)	
Total	61.730	26.369 (42.7)	35.361	51.817	25.448 (72.0)	

### Over-treatment with ACT among malaria negative patients

Frequency of over-prescription of ACT was investigated by assessing the proportion of malaria negative patients treated with ACT. Table 1 shows that 72.0 % (25,448/35,361) of patients with negative malaria results were treated with ACT. Prescription of ACT to malaria negative patients was similar in patients seeking care at health facilities with and without laboratory facilities (72.0 %; 23,164/32,185 versus 71.9 %; 2284/3176 in health facilities with and without laboratory respectively, p = 0.899), but was higher in health facilities located in the central region of the country (81.1 %; 10,673/13,151) as compared to those in the northern (72.4 %; 5247/7248) and southern (63.7 %; 9528/14,962) regions (p = 0.000). Prescription of ACT to malaria negative patients was also higher in urban areas (88.7 %; 14,223/16,005) as compared to rural areas, (58.0 %; 11,225/19,356), respectively (p = 0.000) (Fig. 1).

**Fig. 2:**
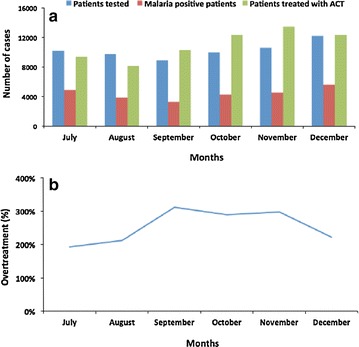
Monthly variation of the frequency of patients tested, confirmed and treated for malaria. **a** Patients tested and confirmed for malaria and treated with ACT and **b** monthly variation in the proportion of patients over-treated with ACT

Figure 2a, b show that the lowest frequency of ACT prescription to malaria negative patients was observed during July and August, with a slight increase from September to December.

### Profile of stock-out of RDT and its impact on prescription of ACT

Stock-out of RDT and its impact on ACT prescription was also assessed. During the period under investigation, a total of six (27.3 %) health facilities registered stock-out of RDT. Table 2 shows that the duration of stock-out in these health facilities varied from <1 month up to three consecutive months.

**Table 2:** List of health facilities with stock out of RDT, the corresponding period and type of RDT stock out

Months between July and December 2011 in which stock out of RDT was reported, month	Type of stock out of RTD	Number of patients tested with RDT	Number of patients treated with ACT
Health facility with stock out of RDT # 1			
July and August	Total^a^	0	268
September and December	Partial^b^	311	971
Health facility with stock out of RDT # 2			
September, October and December	Total	0	287
Health facility with stock out of RDT # 3			
December	Total	0	80
Health facility with stock out of RDT # 4			
July	Total	0	360
Health facility with stock out of RDT # 5			
July	Partial	50	210
August	Total	0	210
September and October	Partial	50	210
November	Total	0	210
Health facility with stock out of RDT # 6			
July and August	Total	0	283
September and December	Partial	233	386

^a^Total stock out of RDT = duration of stock out was 30 days (entire month)

^b^Partial stock out of RDT = duration of stock out was <30 days

Table 2 also shows that in health facilities with partial stock-out, the number of patients who received ACT was much higher than the number of patients who were tested for malaria. Of remark, during the periods of total stock-out of RDT, no patient was tested for malaria and all suspected cases were empirically treated with ACT.

### Profile of stock-out of ACT and its impact on prescription of ACT

Table 2 indicates that stock-out of ACT was reported in three (13.6 %), out of 22 health facilities sampled. Table 3 shows that during periods of partial stock-out of ACT, the number of patients who received ACT was lower than those who were malaria positive. Of note, during periods of total stock-out of ACT, none of the patients with malaria positive results received ACT and all of them were referred to the nearby health facility to receive their anti-malarial treatment.

**Table 3:** Patients treated with ACT during periods of stock out of ACT

Health facility, month	Type of stock out of ACT	A—number of patients tested for malaria	B—number of patients with malaria pos. result, n (B/A %)	C—number of patients treated with ACT, n (C/B %)
Health facility with stock out of ACT # 1				
August	Total^a^	1367	524 (38.3)	0
Health facility with stock out of ACT # 2				
October and November	Partial^b^	645	361 (56.0)	180 (49.9)
Health facility with stock out of ACT # 3				
September	Partial	1080	320 (29.6)	140 (43.7)

^a^Total stock out of ACT = duration of stock out was 30 days (entire month)

^b^Partial stock out of ACT = duration of stock out was <30 days

## Discussion

In 2011 Mozambique adopted the new WHO guidelines for the management of malaria and stipulated that prescription of ACT for the treatment of uncomplicated malaria should be guided and based on laboratory confirmation using either microscopy or RDT as opposed to presumptive diagnosis and treatment. Health care workers’ adherence to this guideline has never been assessed in Mozambique and data from other sub-Saharan countries are conflicting [9, 10, 18, 19], suggesting that prescription practices for ACT is country specific.

This is the first study investigating health care workers’ adherence to these guideline in Mozambique. Results of this study showed that ACT was prescribed to 72.0 % (25,448/35,361) of patients with negative malaria results. This suggests that despite the availability of laboratory testing using either RDT or microscopy in Mozambique, health care workers’ adherence to the guideline for malaria treatment is poor and the disease is largely treated presumptively. Similar results were found in other sub-Saharan Africa countries where malaria is endemic [10, 13, 32]. A study conducted in Zambia demonstrated that 84 % of patients who received ACT were malaria negative [16] and another study conducted in Malawi found that ACT was prescribed to 58 % of febrile patients with negative RDT results [10]. As previously mentioned, over-treatment with ACT is expensive, and leads to unnecessary wastage of drugs, increased morbi-mortality associated with misdiagnosis of treatable non-malaria febrile illnesses and rapid acquisition of drug resistance [6]. Adherence to the new protocol for malaria management is considered a cost effective intervention, as a study conducted in Kenya demonstrated that correct management of malaria can save up to 60 % of costs associated with malaria treatment [14].

Concern on the over treatment of ACT has recently increased, because the burden of malaria is declining in many sub-Saharan Africa countries, including in Mozambique [20, 21, 25], and as a consequence, the likelihood of treating malaria negative with ACT may increase.

While the results of this study suggest that RDT results are underutilized in Mozambique, the underlying reasons for poor uptake and utilization of malaria RDTs results were not addressed in this study and merit further investigation. However, socio-behavioural studies from other countries demonstrate that patients’ expectations to receive anti-malarial treatment along with a lack of skills to deal with patients’ expectations in the presence of negative result places a strong psychological pressure on clinicians [33–35]. This suggests that community education on management of febrile illness is also critical. Other reasons for the overtreatment of ACT described in previous studies conducted in other sub-Saharan Africa countries are: (i) clinicians’ resistance to adhere to the new guidelines, because presumptive diagnosis was a common and accepted practice for many years, (ii) lack of trust in the national laboratory systems, (iii) fear of false negative test results [16, 36], and (iv) lack of epidemiological information on the alternative diagnosis of acute fever [6]. Because of this, there is an urgent need of studies to investigate the aetiology of fever of unknown origin in sub-Saharan Africa.

It’s well documented that comprehensive training, periodic refresher courses and regular supervision visits leads to improvement of clinicians’ adherence to the new guidelines for malaria management. In fact, recent studies conducted in Tanzania demonstrated that adherence to new malaria guidelines was enhanced by comprehensive training, education and supervision [9, 37]. Another study conducted in Ghana found similar results [38]. Implementation of quality control measures is also important, and previous studies demonstrated that the expansion of external quality control programmes for RDT and microscopy improved clinicians’ confidence of results [9, 39, 40].

Data from this study showed that stock-out of RDT was a common finding and resulted in increase in the frequency of presumptive ACT prescription. These findings are similar to those from a study conducted in Benin in 2010, which found that stock-out of RDT occurred in up to 38.5 % of health facilities and influenced prescription of ACT [41]. This suggests that improvement in the supply chain for ACT is critical to minimize over-prescription of ACT.

In this study, adherence to the new protocol for malaria management was lower in the central part of the country, suggesting that health care workers’ prescription practices varies across the country. Over-prescription was also slightly higher in rural areas as compared to urban areas. This might be due to higher literacy and better access to information among clinicians working in urban settings, but additional research is needed to investigate this.

Of note, the quality of medical records is a limiting factor when conducting retrospective studies. To minimize some of these problems, the followings strategies were implemented during this study, (i) comprehensive training of all staff who were involved in data collection, (ii) random selection of questionnaires for quality checks and (iii) only recent data, corresponding to the period between July and December 2011 were collected, since the risk of loosing medical records increases as the storage time increases. Despite these efforts, common errors may still have occurred. The Ministry of Health recently launched its strategy to improve the quality of routine data.

## Conclusions

Overall, the findings of this study showed that clinicians’ adherence to the new WHO guidelines for malaria management in Mozambique are poor and varies by regions of the country. Stock out of RDT is a strong contributing factor for the presumptive treatment with ACT. This study suggests that the roll out of RDT should be accompanied by continuous training, frequent refresh courses, regular supervision, community education, implementation of quality assurance schemes and improvement of supply chain for RDT.

## References

[1] Barat L, Chipipa J, Kolczak M, Sukwa T (1999). Does the availability of blood slide microscopy for malaria at health centers improve the management of persons with fever in Zambia?. Am J Trop Med Hyg.

[2] Bojang KA, Obaro S, Morison LA, Greenwood BM (2000). A prospective evaluation of a clinical algorithm for the diagnosis of malaria in Gambian children. Trop Med Int Health.

[3] Chandramohan D, Jaffar S, Greenwood B (2002). Use of clinical algorithms for diagnosing malaria. Trop Med Int Health.

[4] Luxemburger C, Nosten F, Kyle DE, Kiricharoen L, Chongsuphajaisiddhi T, White NJ (1998). Clinical features cannot predict a diagnosis of malaria or differentiate the infecting species in children living in an area of low transmission. Trans R Soc Trop Med Hyg.

[5] Opoka RO, Xia Z, Bangirana P, John CC (2008). Inpatient mortality in children with clinically diagnosed malaria as compared with microscopically confirmed malaria. Pediatr Infect Dis J.

[6] Joshi R, Colford JM, Reingold AL, Kalantri S (2008). Nonmalarial acute undifferentiated fever in a rural hospital in central India: diagnostic uncertainty and overtreatment with antimalarial agents. Am J Trop Med Hyg.

[7] WHO. Guidelines for the treatment of malaria, 2nd ed. Geneva, World Heal Organization; 2010. 25473692

[8] Batwala V, Magnussen P, Hansen KS, Nuwaha F (2011). Cost-effectiveness of malaria microscopy and rapid diagnostic tests versus presumptive diagnosis: implications for malaria control in Uganda. Malar J.

[9] D’Acremont V, Kahama-Maro J, Swai N, Mtasiwa D, Genton B, Lengeler C (2011). Reduction of anti-malarial consumption after rapid diagnostic tests implementation in Dar es Salaam: a before-after and cluster randomized controlled study. Malar J.

[10] Chinkhumba J, Skarbinski J, Chilima B, Campbell C, Ewing V, San Joaquin M (2010). Comparative field performance and adherence to test results of four malaria rapid diagnostic tests among febrile patients more than five years of age in Blantyre, Malawi. Malar J.

[11] WHO. World malaria report 2012. Geneva: World Health Organization; 2012. p. 27–58.

[12] Wilson ML (2013). Laboratory diagnosis of malaria: conventional and rapid diagnostic methods. Arch Pathol Lab Med.

[13] Reyburn H, Mbakilwa H, Mwangi R, Mwerinde O, Olomi R, Drakeley C (2007). Rapid diagnostic tests compared with malaria microscopy for guiding outpatient treatment of febrile illness in Tanzania: randomised trial. BMJ.

[14] Zurovac D, Larson BA, Akhwale W, Snow RW (2006). The financial and clinical implications of adult malaria diagnosis using microscopy in Kenya. Trop Med Int Health.

[15] Diggle E, Asgary R, Gore-Langton G, Nahashon E, Mungai J, Harrison R (2014). Perceptions of malaria and acceptance of rapid diagnostic tests and related treatment practises among community members and health care providers in Greater Garissa, North Eastern Province, Kenya. Malar J.

[16] Manyando C, Njunju EM, Chileshe J, Siziya S, Shiff C (2014). Rapid diagnostic tests for malaria and health workers’ adherence to test results at health facilities in Zambia. Malar J.

[17] Yukich JO, Bennett A, Albertini A, Incardona S, Moonga H, Chisha Z (2012). Reductions in artemisinin-based combination therapy consumption after the nationwide scale up of routine malaria rapid diagnostic testing in Zambia. Am J Trop Med Hyg.

[18] Thiam S, Thior M, Faye B, Ndiop M, Diouf ML, Diouf MB (2011). Major reduction in anti-malarial drug consumption in Senegal after nation-wide introduction of malaria rapid diagnostic tests. PLoS One.

[19] Bilal JA, Gasim GI, Abdien MT, Elmardi KA, Malik EM, Adam I (2015). Poor adherence to the malaria management protocol among health workers attending under-five year old febrile children at Omdurman hospital, Sudan. Malar J.

[20] Moonasar D, Nuthulaganti T, Kruger PS, Mabuza A, Rasiswi ES, Benson FG (2012). Malaria control in South Africa 2000–2010: beyond MDG6. Malar J.

[21] mal ERACGoD. Diagnostics: a research agenda for malaria eradication: diagnoses and diagnostics. PLoS Med 2011;8:e1000396. 10.1371/journal.pmed.1000396 PMC3026696 21311583

[22] ter Kuile FO, Terlouw DJ, Phillips-Howard PA, Hawley WA, Friedman JF, Kariuki SK (2003). Reduction of malaria during pregnancy by permethrin-treated bed nets in an area of intense perennial malaria transmission in western Kenya. Am J Trop Med Hyg.

[23] Mabaso ML, Sharp B, Lengeler C (2004). Historical review of malarial control in southern African with emphasis on the use of indoor residual house-spraying. Trop Med Int Health.

[24] Andrianasolo RL, Rakotoson J, Rakotoarivelo RA, Andriamahenina R, Randria Mamy JD, Rapelanoro Rabenja F (2012). Adhesion of physicians to the national malaria policy: situation in Antananarivo (Madagascar), 5 years after the policy revision (in French). Med Sante Trop.

[25] Sharp BL, Kleinschmidt I, Streat E, Maharaj R, Barnes KI, Durrheim DN (2007). Seven years of regional malaria control collaboration—Mozambique, South Africa, and Swaziland. Am J Trop Med Hyg.

[26] O’Meara WP, Mangeni JN, Steketee R, Greenwood B (2010). Changes in the burden of malaria in sub-Saharan Africa. Lancet Infect Dis.

[27] Barnes KI, Chanda P, Ab Barnabas G (2009). Impact of the large-scale deployment of artemether/lumefantrine on the malaria disease burden in Africa: case studies of South Africa, Zambia and Ethiopia. Malar J.

[28] Blumberg L, Frean J (2007). Malaria control in South Africa—challenges and successes. S Afr Med J.

[29] Instituto Nacional de Estatística. Resultados Definitivos do Censo Geral da Populacional e Habitação—2007; Disponivel em. http://www.ine.gov.mz/censo2007. Acedido em 22 de Maio de 2013.

[30] Langa FJL. Atlas do perfil habitacional de Moçambique (1997 a 2007), Uma abordagem do SIG; Tese de Mestrado, Universidade de Lisboa. 2010;66-69.

[31] D TA. Normas de Tratamento da Malaria em Moçambique (MISAU D ed.). Moçambique 2011;11–12:36.

[32] Mwanziva C, Shekalaghe S, Ndaro A, Mengerink B, Megiroo S, Mosha F (2008). Overuse of artemisinin-combination therapy in Mto wa Mbu (river of mosquitoes), an area misinterpreted as high endemic for malaria. Malar J.

[33] Gilson L, Alilio M, Heggenhougen K (1994). Community satisfaction with primary health care services: an evaluation undertaken in the Morogoro region of Tanzania. Soc Sci Med.

[34] Ofori-Adjei D, Arhinful DK (1996). Effect of training on the clinical management of malaria by medical assistants in Ghana. Soc Sci Med.

[35] Chandler CI, Whitty CJ, Ansah EK (2010). How can malaria rapid diagnostic tests achieve their potential? A qualitative study of a trial at health facilities in Ghana. Malar J.

[36] Msellem MI, Martensson A, Rotllant G, Bhattarai A, Stromberg J, Kahigwa E (2009). Influence of rapid malaria diagnostic tests on treatment and health outcome in fever patients, Zanzibar: a crossover validation study. PLoS Med.

[37] Masanja MI, McMorrow M, Kahigwa E, Kachur SP, McElroy PD (2010). Health workers’ use of malaria rapid diagnostic tests (RDTs) to guide clinical decision making in rural dispensaries, Tanzania. Am J Trop Med Hyg.

[38] Baiden F, Owusu-Agyei S, Okyere E, Tivura M, Adjei G, Chandramohan D, Webster J (2012). Acceptability of rapid diagnostic test-based management of malaria among caregivers of under-five children in rural Ghana. PLoS One.

[39] Hopkins H. Effectiveness and safety of training in fever case management and RDT use at health centres in Uganda. In: American Society of Tropical Medicine & Hygiene. (57th annual meeting, New Orleans);2008.

[40] Williams HA, Causer L, Metta E, Malila A, O’Reilly T, Abdulla S (2008). Dispensary level pilot implementation of rapid diagnostic tests: an evaluation of RDT acceptance and usage by providers and patients—Tanzania, 2005. Malar J.

[41] Programe National de Lutte Contre le Paludisme (PNLP). Bulletin du systeme d’information de routine sur le paludisme au Bénin (InFO S ed.). Ministère de la santé Bulletin no 9. 2010;1–4.

